# Intravenous Lipid Emulsion Therapy for Severe Emamectin Benzoate Poisoning: A Case Report

**DOI:** 10.7759/cureus.110830

**Published:** 2026-06-14

**Authors:** Ahmed A Al-Adhroey, Mohammed A Al-Mahdi, Ahmed Y Al-Waisi

**Affiliations:** 1 Gastroenterology and Hepatology, Faculty of Medicine and Health Sciences, Sana'a University, Sana'a, YEM; 2 Internal Medicine, Al-Thawra Modern General Hospital, Sana'a, YEM

**Keywords:** acute coma, acute pesticide poisoning, emamectin benzoate, intravenous lipid emulsion (ile), lipophilic toxicity, toxicology

## Abstract

Emamectin benzoate poisoning is an uncommon but potentially life-threatening cause of neurocardiovascular toxicity. We report the case of a 22-year-old man who presented with coma, respiratory failure, and refractory shock after intentional ingestion of emamectin benzoate. Conventional supportive measures were instituted, but clinical instability persisted until rescue intravenous lipid emulsion (ILE) was administered. Hemodynamic recovery followed the first dose, and neurological improvement was observed after the second dose. This case suggests a possible role for ILE as adjunctive rescue therapy in selected patients with severe emamectin benzoate poisoning after conventional supportive measures have failed.

## Introduction

Emamectin benzoate is an agricultural insecticide belonging to the avermectin family of macrocyclic lactones. While instances of intentional and accidental poisonings have been rising globally, particularly in low- and middle-income countries due to widespread agricultural availability, severe human toxicity remains relatively rare but clinically challenging. In humans, the molecular mechanism of action primarily involves interactions with gamma-aminobutyric acid (GABA) receptors in the central nervous system (CNS), specifically at high doses where the toxin crosses the blood-brain barrier [1,2]. Unlike in invertebrates, where avermectins target glutamate-gated chloride channels (which are absent in humans), toxicity in mammalian systems is driven by the potentiation of GABAergic neurotransmission [2]. This stimulation of GABA release and receptor binding leads to neuronal hyperpolarization, which clinically translates into severe neurological and respiratory manifestations [1,2]. The global incidence of severe emamectin benzoate toxicity is low, but clinical outcomes depend heavily on specific prognostic factors such as the volume of ingestant, co-ingested surfactants, and delayed presentation to healthcare facilities. Acute ingestion typically manifests with early gastrointestinal effects, including nausea, persistent vomiting, abdominal pain, and diarrhea, in up to 60% of patients [1]. In severe cases, CNS depression is observed in up to 80% of patients, and complications like respiratory failure or shock can affect 31% of severe ingestions [1,3]. While mild exposures often resolve with basic interventions, severe poisonings carry an average mortality rate of 6.7%, which can spike up to 40% in critical instances due to severe metabolic acidosis and asphyxia from vomitus [1,3]. Currently, the available management protocol is strictly supportive and symptomatic, focusing on airway protection, mechanical ventilation, and hemodynamic stabilization, as no specific antidote is available [1,2,4]. Intravenous lipid emulsion (ILE) has been utilized in selected poisonings involving highly lipophilic compounds and may reduce tissue toxicity through sequestration of the toxin within an intravascular lipid phase (the "lipid sink" effect) [5-8]. However, published clinical experience with ILE in emamectin benzoate poisoning remains extremely limited. Here, we report a case of severe emamectin benzoate poisoning in which clinical recovery followed administration of ILE. Given the limited evidence base, this observation should be interpreted cautiously and should not be generalized without further clinical study [1,2].

## Case presentation

A 22-year-old man with no significant past medical history, weighing 65 kg, was brought to the emergency department approximately three hours after intentional ingestion of a full 100 mL commercial container of 5% emamectin benzoate emulsifiable concentrate (EC) pesticide formulation. On arrival, he was unconscious, with a Glasgow Coma Scale (GCS) score of 6/15 (E1V1M4), bilaterally dilated but reactive pupils, generalized hypotonia, and absent deep tendon reflexes. His blood pressure was 60/40 mmHg, pulse rate 138 beats/min, respiratory rate (RR) 10 breaths/min, and oxygen saturation 83% on room air. The patient underwent immediate endotracheal intubation and was placed on mechanical ventilation using synchronized intermittent mandatory ventilation with volume control (SIMV-VC) mode with the following initial parameters: tidal volume of 400 mL (6 mL/kg based on ideal body weight), an RR of 14 breaths/min, positive end-expiratory pressure (PEEP) of 5 cmH_2_O, and an initial fraction of inspired oxygen (FiO_2_) titrated at 60% to maintain target oxygen saturation. No sedative agents were administered because the patient was already unconscious upon presentation, and withholding sedation allowed accurate serial neurological assessment throughout the clinical course. During the initial resuscitation phase, a right subclavian central venous catheter was inserted, revealing a low central venous pressure (CVP) of 3 cmH_2_O, suggestive of significant intravascular volume depletion. Due to the severity of poisoning and the highly lipophilic nature of the ingested compound, gastric lavage and activated charcoal were administered despite presentation beyond the conventional decontamination window. Initial investigations revealed leukocytosis, hypoxemia, severe mixed respiratory-metabolic acidosis, hypokalemia, mild hypocalcemia, and an elevated serum lactate level of 4 mmol/L, while renal and hepatic functions remained preserved (Table 1).

**Table 1:** Clinical, hemodynamic, arterial blood gas, and laboratory findings during intensive care unit admission following severe emamectin benzoate poisoning ABG: arterial blood gas; BPM: beats per minute; GCS: Glasgow Coma Scale; HCO_3_: bicarbonate; PaCO_2_: partial pressure of carbon dioxide; PaO_2_: partial pressure of oxygen; pH: potential of hydrogen; SpO_2_: peripheral oxygen saturation; WBC: white blood cell count; ALT: alanine aminotransferase; AST: aspartate aminotransferase; CPK: creatine phosphokinase Note: All normal reference ranges provided in this table are derived from the local reference intervals established by our hospital's central laboratory.

Parameter	Unit	Day 1	Day 2	Day 3	Normal range
GCS Score	-	6/15	15/15	15/15	15/15
Systolic Blood Pressure	mmHg	60	110	120	100-140
Diastolic Blood Pressure	mmHg	40	70	80	60-90
Pulse Rate	bpm	138	74	80	60-100
SpO_2_ (Room Air)	%	83	96	98	95-100
Arterial pH	-	7.28	7.41	7.40	7.35-7.45
PaCO_2_	mmHg	48	39	40	35-45
PaO_2_	mmHg	54	92	95	80-100
HCO_3_	mmol/L	18	24	24	22-26
Base Deficit	mEq/L	7.6	0.2	0	-2 to 2
Serum Lactate	mmol/L	4.0	1.0	1.0	0.5-2.0
Serum Potassium	mEq/L	3.1	4.0	4.2	3.5-5.1
Serum Sodium	mEq/L	142	140	142	136-145
Random Blood Sugar	mg/dL	123	110	100	70-140
Serum Calcium	mg/dL	7.8	8.9	9.2	8.5-10.5
WBC Count	cells/μL	17,000	12,000	8,000	4,000-11,000
Triglycerides	mg/dL	131	190	140	<150
Serum Amylase	U/L	29	36	40	30-110
Serum Lipase	U/L	43	51	45	13-60
ALT	U/L	40	41	35	<40
AST	U/L	31	24	28	<40
Serum Creatinine	mg/dL	0.9	1.1	1.0	0.7-1.3
CPK	U/L	184	340	180	22-198
Urine Output	mL/kg/hr	0.9	1.0	1.0	0.5-1.5

Aggressive fluid resuscitation was initiated with 2 L of isotonic crystalloid (0.9% sodium chloride). However, despite adequate volume replacement, the patient remained profoundly hypotensive with no meaningful neurological improvement. Approximately one hour after emergency department admission (four hours after ingestion), the patient was transferred and admitted to the intensive care unit (ICU). Upon ICU admission, standard supportive care measures were continued. Maintenance intravenous fluids were continued using 0.9% sodium chloride at a continuous rate of 100 mL/hr. Electrolyte imbalances were corrected, including continuous intravenous replacement with 20 mEq of potassium chloride (KCl) per liter of maintenance fluid to correct hypokalemia, and a slow infusion of 1 g of 10% calcium gluconate for hypocalcemia. Stress ulcer prophylaxis was initiated with intravenous omeprazole (40 mg q24h), alongside mechanical deep vein thrombosis (DVT) prophylaxis. Continuous, hemodynamic and multi-parameter monitoring was established to guide further resuscitation, consisting of continuous 5-lead electrocardiography (ECG) for heart rate and rhythm surveillance, continuous pulse oximetry (SpO_2_), non-invasive blood pressure (NIBP) cycling every 10-15 minutes during unstable phases, and hourly CVP measurements via the subclavian catheter. Renal perfusion was closely monitored through strict, hourly urine output measurement through an indwelling Foley catheter, which showed a stable output of 0.9 mL/kg/hour over the first 24 hours. Continuous intravenous norepinephrine bitartrate infusion was initiated at 0.2 mcg/kg/min and progressively titrated every 10 min to maintain a mean arterial pressure (MAP) above 65 mmHg. The dose was escalated to a peak of 0.8 mcg/kg/min, yet the patient continued to exhibit refractory, vasopressor-dependent shock, with blood pressure remaining approximately 80/50 mmHg and only minimal improvement in CVP. A second vasopressor was not added at that stage, and the team prioritized ongoing resuscitation and rescue adjunctive therapy, based on local clinical judgment and concern for worsening vasoconstriction.

Given the persistent hemodynamic instability, severe mixed respiratory-metabolic acidosis, elevated serum lactate, marked lipophilicity of emamectin benzoate, and the absence of a specific antidote, rescue therapy with ILE was initiated nine hours after ingestion (five hours after ICU admission). Because the standard 20% ILE formulation was unavailable owing to supply limitations in Yemen, Celepid™ 10% (Otsuka Pharmaceutical Factory, Inc., Naruto, Tokushima, Japan) was used as an alternative. The administered volume and infusion rate were adjusted to approximate the intended lipid dose based on available toxicology guidance. Celepid™ 10% is a sterile, non-pyrogenic fat emulsion supplied in a 500 mL glass container. Each 100 mL contains 10.08 g soybean oil USP, 1.20 g egg lecithin, and 2.25 g glycerol USP in water for injection BP. The formulation provides 1100 kcal/L and has an osmolarity of 300 mOsm/L. Based on the patient's body weight of 65 kg, the initial adjusted bolus dose of 3 mL/kg of Celepid™ 10% equated to a total volume of 195 mL, administered intravenously over 15 min. This was followed immediately by a continuous maintenance infusion of 0.5 mL/kg/min for 30 minutes, delivering an additional volume of 975 mL (calculated as 32.5 mL/min over 30 minutes). A significant hemodynamic response became evident within the following hours. By 12 hours after ingestion, blood pressure had improved to 100/80 mmHg, heart rate decreased to 93 beats/min, CVP increased to 8 cmH_2_O, and serum lactate normalized to 1 mmol/L. These improvements allowed initiation of a gradual norepinephrine bitartrate tapering at a rate of 0.1 mcg/kg/min every hour. Despite rapid hemodynamic stabilization, neurological recovery lagged behind, and the patient remained deeply stuporous. A second bolus dose of ILE was therefore delayed until 14 hours after ingestion (five hours after the first bolus) to allow time for the initial lipid load to exert its effect, confirm sustained hemodynamic stability, and reduce the potential risk of acute hypertriglyceridemia or fat overload syndrome. After persistent neurological impairment was confirmed despite circulatory improvement, a second adjusted bolus dose of 3 mL/kg (195 mL) of Celepid™ 10% was administered over 30 minutes. The cumulative volume of 10% ILE administered across all three steps was 1365 mL. Following the second ILE administration, neurological status improved progressively. At 18 hours after ingestion, the patient demonstrated spontaneous eye opening and purposeful motor responses, while norepinephrine requirements had decreased to 0.2 mcg/kg/min. Intravenous fluids were discontinued after adequate hemodynamic targets had been achieved. By 20 hours after ingestion, the patient was awake, following commands, and considered suitable for ventilator weaning. At 22 hours after ingestion, he was successfully extubated, with complete neurological recovery (GCS 15/15). He was fully oriented and maintained stable spontaneous respiration. Norepinephrine was discontinued without rebound hypotension. At 24 hours after ingestion, he remained fully conscious and hemodynamically stable, with a blood pressure of 110/70 mmHg and normal oxygenation. Throughout hospitalization, serial safety monitoring demonstrated only a mild increase in triglyceride levels from 131 to 190 mg/dL, while serum amylase and lipase remained within normal reference ranges. No evidence of acute pancreatitis or other lipid-related adverse effects was observed. On day 3, the patient was clinically stable and successfully discharged from the ICU to the general medical ward for a 24-hour step-down observation period. Following complete clinical stability and a formal psychiatric assessment, he was safely discharged home from the hospital on day 4 without apparent neurological or systemic sequelae. Before discharge, the patient was referred for long-term psychiatric follow-up to address the underlying self-harm intent. A detailed counseling session was also conducted with his family members. They were educated about the toxicity of household agrochemicals, the importance of removing toxic substances from the home, and strategies to restrict access to pesticides in the domestic environment.

Baseline clinical and laboratory findings during the (ICU) stay are summarized in Table 1, and the chronological sequence of clinical events, hemodynamic parameters, neurological progression, and therapeutic interventions is presented in Table 2.

**Table 2:** Chronological timeline of clinical course, hemodynamic parameters, and therapeutic interventions from time of ingestion to hospital discharge BP: blood pressure measured non-invasively using NIBP (non-invasive blood pressure monitoring); HR: heart rate; RR: respiratory rate; SpO_2_: peripheral oxygen saturation; CVP: central venous pressure measured via right subclavian central venous catheter; GCS: Glasgow Coma Scale; E: eye response, V: verbal response, M: motor response; VT: intubated patient (no verbal response); MAP: mean arterial pressure; ILE: intravenous lipid emulsion (10% formulation); norepinephrine doses are expressed in mcg/kg/min and were titrated according to hemodynamic response; lactate values are arterial serum lactate levels; mechanical ventilation was delivered using SIMV-VC mode; gastric decontamination included gastric lavage followed by activated charcoal administration; all times are referenced from estimated time of ingestion (0 hour).

Time from ingestion	GCS	Hemodynamic parameters	Key interventions and clinical events
0 hour	-	-	Intentional ingestion of approximately 100 mL of 5% emamectin benzoate EC.
3 hours (ED arrival)	E1 V1 M4 (GCS 6/15)	BP 60/40 mmHg, HR 138 bpm, RR 10/min, SpO₂ 83%, CVP 3 cmH₂O	Endotracheal intubation and mechanical ventilation (SIMV-VC). Gastric lavage and activated charcoal administered. Right subclavian central venous catheter inserted. Initial fluid resuscitation with 2 L 0.9% normal saline.
4 hours	E1 VT M4	BP 60/40 mmHg, CVP 3 cmH₂O, lactate 4 mmol/L	No response to initial resuscitation. Norepinephrine started at 0.2 mcg/kg/min and titrated to maintain MAP >65 mmHg. Maintenance fluids 100 mL/h.
5 hours	E1 VT M4	BP 80/50 mmHg, CVP 4 cmH₂O, norepinephrine 0.8 mcg/kg/min	Persistent vasopressor-dependent shock despite aggressive resuscitation.
9 hours	E1 VT M4	BP 80/50 mmHg, HR 110 bpm, CVP 5 cmH₂O, lactate 3 mmol/L	First ILE 10%: 3 mL/kg bolus over 15 min then 0.5 mL/kg/min infusion for 30 minutes. Maintenance fluids reduced to 50 ml/h.
12 hours	E2 VT M4	BP 100/80 mmHg, HR 93 bpm, CVP 8 cmH₂O, lactate 1 mmol/L	Hemodynamic improvement after ILE. Norepinephrine tapering initiated.
14 hours	E2 VT M4	BP 110/80 mmHg, HR 84 bpm, norepinephrine 0.6 mcg/kg/min	Second ILE dose administered due to persistent depressed consciousness.
18 hours	E3 VT M5	CVP 8 cmH₂O, norepinephrine 0.2 mcg/kg/min, lactate 1 mmol/L	Progressive neurological recovery. IV fluids discontinued.
20 hours	E4 VT M6	Norepinephrine 0.1 mcg/kg/min	Awake, following commands. Preparing for ventilator weaning.
22 hours	E4 V5 M6 (GCS 15/15)	Hemodynamically stable off vasopressors	Successfully extubated. Fully conscious and oriented.
24 hours	E4 V5 M6 (GCS 15/15)	BP 110/70 mmHg	Full neurological and hemodynamic stability achieved. No rebound hypotension
Day 2	E4 V5 M6 (GCS 15/15)	Stable vital signs and laboratory parameters as shown in Table 1	Clinically stable under close monitoring in ICU with no new events.
Day 3	E4 V5 M6 (GCS 15/15)	Fully stable; as per Table 1	Completely stable; transferred to general medical ward for further 24-hour observation.
Day 4	E4 V5 M6 (GCS 15/15)	Fully conscious, vitally and systematically stable.	Discharged home in good clinical condition with advice for psychiatric follow-up.

## Discussion

Severe emamectin benzoate poisoning is rare, but potentially fatal. Reported manifestations include coma, respiratory failure, and cardiovascular instability following large ingestion [1-4,9]. According to published clinical literature and retrospective poisoning cohorts, neurological manifestations are the most predominant features, with altered consciousness or coma occurring in approximately 60%-70% of symptomatic cases [1,4]. Conversely, severe hemodynamic instability and refractory shock are less frequent, reported in about 20%-30% of poisonings, and are typically associated with massive ingestions or co-ingested surfactant vehicles [1,4]. ​The presentation in this patient was consistent with previously described severe toxicity and required ICU admission. ILE was administered because the patient remained unstable despite standard supportive management described previously, and emamectin benzoate is highly lipophilic. The proposed mechanism is the "lipid sink" or "lipid shuttle" effect, whereby an expanded lipid phase reduces the free concentration of lipophilic toxin and promotes redistribution away from target tissues [5-8]. Evidence supporting ILE is strongest in local anesthetic systemic toxicity, although it has also been used as salvage therapy in overdoses involving other lipophilic agents [5-8,10].

In the present case, hemodynamic recovery began within hours of ILE administration and was followed by neurological improvement after the second dose. Nevertheless, supportive care, ongoing metabolism, and toxin clearance may also have contributed to the observed recovery. Although the temporal relationship between ILE administration and clinical improvement is notable, the specific effect of ILE cannot be conclusively distinguished from other concurrent factors. ​Beyond ILE, advanced extracorporeal life support and detoxification techniques have gathered increasing interest in severe, refractory poisonings. Specifically, extracorporeal hemoadsorption technologies, such as the CytoSorb® device, utilize highly biocompatible polystyrene-divinylbenzene copolymer beads to capture and eliminate hydrophobic or protein-bound hydrophobic toxins, cytokines, and xenobiotics from the bloodstream via concentration-dependent hydrophobic interactions. Given that emamectin benzoate possesses a high molecular weight, strong protein binding, and marked lipophilicity, conventional hemodialysis or hemofiltration are generally ineffective for its clearance. In such clinical scenarios, targeted hemoadsorption or advanced lipid-augmented therapies - such as single-pass lipid dialysis, which has shown promise in enhancing the clearance of closely related avermectin toxicities - could theoretically serve as an adjunctive or alternative salvage modality to accelerate toxin clearance and mitigate systemic inflammatory cascades [11]. However, clinical evidence for these advanced extracorporeal modalities in pure emamectin benzoate poisoning remains extremely limited and largely theoretical. Furthermore, the deployment of such advanced extracorporeal techniques is highly constrained in resource-limited settings, such as our center in Yemen, due to prohibitive costs, specialized equipment requirements, and supply-chain barriers, which reinforces the clinical value of 10% ILE as an accessible, cost-effective frontline rescue therapy [1,11].

This case report has several limitations. Toxicological confirmation was unavailable, and the diagnosis relied on exposure history, clinical findings, and identification of the ingested product container. In addition, because the ingested preparation was an EC formulation, the contribution of formulation co-components to the observed toxicity cannot be excluded. No adverse effects related to ILE, including pancreatitis or fat overload syndrome, were observed in this patient. Overall, this case adds to the limited literature suggesting that ILE may have a role as rescue therapy in selected patients with severe emamectin benzoate poisoning that is refractory to conventional management.

## Conclusions

Severe emamectin benzoate poisoning may result in life-threatening neurological and cardiovascular toxicity requiring intensive supportive care. In this case, ILE was associated with rapid clinical recovery after conventional measures failed to achieve adequate stabilization. Although causality cannot be established from a single case, this report supports consideration of ILE as a potential rescue therapy in selected patients with severe emamectin benzoate poisoning. Further clinical evidence is needed to clarify its efficacy, optimal timing, and dosing.
